# Portal Vein Thrombosis after the Consumption of Date Seed Powder: A Case Study

**DOI:** 10.1155/2021/6668722

**Published:** 2021-04-17

**Authors:** Mohammad Ali Zakeri, Mohammad Hossein Bagheripour, Marcello Iriti, Mahlagha Dehghan

**Affiliations:** ^1^Social Determinants of Health Research Centre, Rafsanjan University of Medical Sciences, Rafsanjan, Iran; ^2^Non-Communicable Diseases Research Center, Rafsanjan University of Medical Sciences, Rafsanjan, Iran; ^3^Department of General Surgery, Faculty of Medicine, Rafsanjan University of Medical Sciences, Rafsanjan, Iran; ^4^Department of Agricultural and Environmental Sciences, Faculty of Agricultural and Food Sciences, Milan State University, Milan, Italy; ^5^Nursing Research Center, Kerman University of Medical Sciences, Kerman, Iran

## Abstract

Date seeds can be used as ingredients to enhance the nutritional value of some functional foods for human consumption as well as additives in pharmaceutical and cosmetic industries. However, there are no reports on the complications of date seeds after oral consumption. We currently report a patient with no history of gastrointestinal disease, who has been admitted to the hospital with portal vein thrombosis (PVT) and suffered from complications.

## 1. Introduction

The term PVT refers to the complete or partial obstruction of blood flow in the portal vein, due to the presence of thrombus in the vassal lumen, which occurs rarely in the general population [1]. There are numerous factors that can contribute to this disorder, including chronic liver disease, malignancies, processes localized to the epigastrium and hepatobiliary system, and acquired as well as inherited thrombophilia [2]. Acute and chronic PVT has different clinical manifestations. Acute PVT is associated with symptoms such as abdominal pain, diarrhea, abdominal distention, nausea, vomiting, anorexia, fever, lactacidosis, sepsis, and intestinal congestion with ischemia. In contrast, chronic PVT can be completely asymptomatic, or characterized by splenomegaly, pancytopenia, varicose veins, and rarely ascites [3]. Previous studies have shown that some plant products and herbal remedies can cause serious toxic effects and drug interactions that have not been scientifically evaluated [4]. The date palm (*Phoenix dactylifera* L.) rich in valuable nutrients grows in arid and semiarid regions of the world, especially in most Middle-Eastern countries [5].

Date seed is the byproduct of date stoning [6], which is a hard coated seed, usually oblong, ventrally grooved, with a small embryo and weight of 0.5 g to 4 g [6,7]. A study conducted in Iran has shown that the date seeds on an average contain 4.84% protein, 12.22% fat, 27.58% fiber, 80.76% carbohydrates, 1.18% ash, and 1.72% moisture. The date seeds are also a rich source of phenolics and minerals, including iron (Fe), calcium (Ca), copper (Cu), sodium (Na), zinc (Zn), and magnesium (Mg). The main fatty acids of the seed oil are oleic, lauric, palmitic, myristic, linoleic, and stearic acids [8]. Date seeds are also used for therapeutic purposes in some places [9]. Date seeds are used in the caffeine-free drinks in Arab countries, where such a powder is assumed to prevent gastric upset and indigestion [10]. In addition, date seeds have been used in Egyptian folk medicine without scientific evidence for many years to manage liver diseases, diabetes, and gastrointestinal disorders [10]. In the present case study, we report an interesting case, in which PVT has developed after oral consumption of a large amount of date seeds powder. Early diagnosis, proper management, and identification of causal agents may affect the patient's outcome and mortality.

## 2. Case Presentation

An 82-year-old man (height: 170 cm; weight: 62 kg; BMI: 21.4) with a history of diabetes mellitus referred to the hospital because of a generalized abdominal pain with predominance of epigastric pain radiating to the back and an intensity of 8 out of 10 began 4 days ago. He had experienced severe anorexia, nausea, vomiting, constipation, and abdominal distention for 3 days before hospital admission. He was using metformin 500 mg twice a day to control his blood sugar. He had no history of cardiac and liver diseases. He had no problems until 4 days before hospital admission. He had no history of drug or alcohol use and without a thromboembolic disease history, but he had been eating date seeds powder (two tablespoons twice a day) for two weeks to relieve chronic knee joint pain without physician prescription. Absence of bowel sound and generalized tenderness with severe epigastric pain were found on the physical examination. The entire abdomen percussion showed a dull sound, and shifting dullness test was positive for ascites.

His body temperature, oxygen saturation, heart rate, and blood pressure were 36.9°C, 93%, 102 beats a minute, and 111/61 mmHg, respectively. The complete blood count showed the following parameters: white blood cells (WBCs) count 11.9 × 10^9^/L, red blood cell (RBC) count 4.25 mcL, hemoglobin concentration (Hb) 12.2 g/L, hematocrit (Hct) 34.4%, partial thromboplastin time (PTT) 35 sec, prothrombin time (PT) 13 sec, international normalized ratio (INR) 1, bleeding time (BT) 5 minutes, clotting time (CT) 11 minutes, blood urea nitrogen 95 mg/dl, and creatinine 1.5 *μ*mol/L. In addition, the patient test results of factor V Leiden (FAK-tur five LIDE-n) and lupus anticoagulants (LA) were negative.

**Figure 1:**
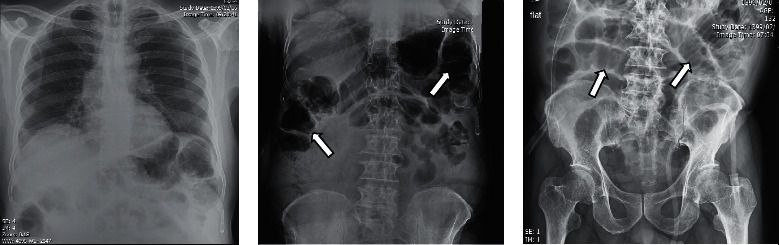
The patient's chest X-ray: (a) no significant finding in chest X-ray; (b) colon is visible; (c) dilated small bowel loops.

**Figure 2:**
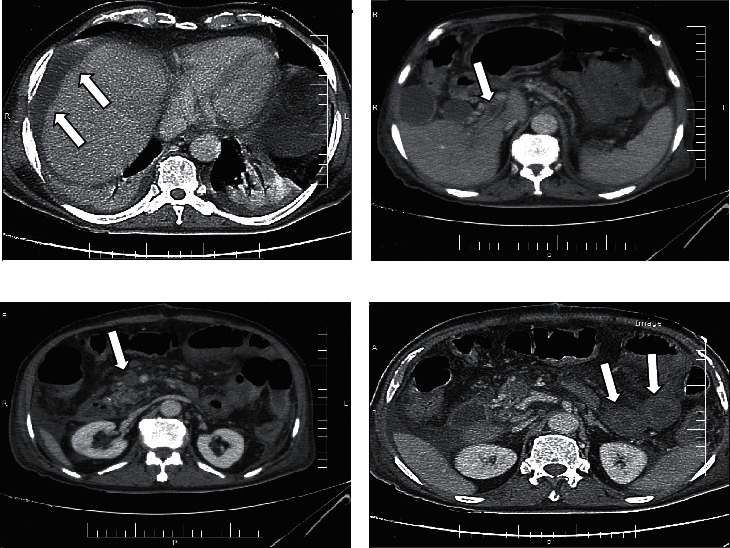
The patient's CT: (a) ascites in chest CT; (b) portal vein thrombosis in abdominal CT; (c) extension of thrombosis in the superior mesenteric vein in abdominal CT; (d) edematous small bowel wall in abdominal CT.

Furthermore, the patient's liver function tests showed alanine transaminase (ALT), 26 IU/L; aspartate transaminase (AST), 17 IU/L; alkaline phosphatase (ALP), 175 IU/L; serum total protein, 5.3 g/dl; serum albumin, 3.1 g/dl; blood sugar, 214 mg/dl; and amylase, 33 IU/L. Ascites fluid sample analysis showed negative culture; albumin, 0.9 g/dl; protein, 1.8 g/dl; WBC, 90 (90% lymph)/mm^3^; RBC, 380/mm^3^; and sugar, 199 mg/dl. Plane abdominal series radiographies and computed tomography (CT) of the abdomen and pelvic were carried out. The radiographic findings corresponded with the ileus of the small bowel (Figure 1). Abdominal CT scan showed portal vein thrombosis with small bowel disseminated edema and ileus (Figure 2).

For exclusion of any other possible diagnosis, like mesenteric venous ischemia, a diagnostic laparoscopy was performed. There was edematous small bowel and dilated colon with no ischemia and pathologic finding in the abdomen and pelvis but the massive ascites. The liver was normal in appearance, and biopsies were taken. Pathological reports of omentum and peritoneum and liver biopsies were normal. Our case study did not have the PVT risk factors, including chronic liver disease, malignancies, processes localized to the epigastrium and hepatobiliary system, and acquired as well as inherited thrombophilia.

In the thorough retrospective survey, we found no other probable reason or specific issue except consumption of date seeds powder. He was treated with conservative management, anticoagulant drugs, and continuous unfractionated heparin 18 U/Kg/h. He got better gradually. Bowel sound was present on the third postoperative day with bisacodyl suppository and lactulose syrup. Hepatic encephalopathy occurred on the 10^th^ postoperative day, and the patient became confused but got better after an increase in lactulose dosage and administration of rifaximin 550 mg twice a day. He felt better over time, and his confusion disappeared completely on the 20^th^ day. He never became icteric. Finally, he was discharged from hospital 25 days later with rivaroxaban 20 mg/day and came back to his normal life within a month. Rivaroxaban treatment was ceased after 3 months. He is well and has normal function by now.

## 3. Discussion

Studies have reported allergy or hypersensitivity to date palm fruit and pollen [11, 12], and date palm fruit is considered as a strong allergen [12]. There are also some concerns about date varieties with high concentration of selenium, which is related to the amount of selenium in the soil [13]. However, to the best of our knowledge, no study has reported the adverse effects of date seeds [14]. We report the first case of PVT after consumption of the date seeds for a long time.

PVT is a vascular disease of the liver that occurs when a blood clot occurs in the hepatic portal vein [1]. PVT was often considered idiopathic in the past, but now PVT can be diagnosed in about 70% of the cases because of prothrombotic abnormalities and a better perception of susceptible clinical conditions [15]. PVT is commonly associated with cirrhosis, abdominal malignancies, and localized inflammation, infection, and prothrombotic disorders. Acute or chronic PVT is often difficult to diagnose. There are major challenges to PVT management strategies, including anticoagulants, thrombolytics, and surgical options. Early diagnosis and proper management have important effects on the rate of mortality [16].

One of the genetic factors that causes PVT is thrombophilia. The factor V G1691A mutation and the prothrombin G20210A mutation are the two most common genetic causes of thrombophilia. However, the effects of these two gene mutations along with other risk factors can increase the occurrence of venous thromboembolism [17]. The JAK2V617F mutation has been described in 17% of the patients with portal and mesenteric venous thrombosis [18]. Prevalence of the JAK2V617F mutation was 1.4% in cirrhotic patients with PVT [19]. Routine screening for the JAK2V617F mutation can be recommended in nonmalignant and noncirrhotic patients with PVT, but not in cirrhotic patients with PVT. The splenomegaly might be closely associated with the JAK2V617F mutation [19]. The prevalence of calreticulin (CALR) mutations in splanchnic vein thrombosis (SVT) varies among studies. Li et al. have shown that the pooled proportion of CALR mutations was 1.59% in PVT patients and 1.82% in PVT patients without JAK2V617F mutation [20]. Based on a meta-analysis by Qi et al., the factor V Leiden (FVL) mutation is associated with an increased risk of PVT among patients with/without cirrhosis. In addition, the prothrombin G20210A mutation is associated with PVT [21].

Examination of the case study showed that the 82-year-old patient had no history of genetic disease or even previous hospitalization. Owing to the fact that genetic diseases and alterations and their complications are appeared at an early age [22], the possibility of genetic problems in this patient is unlikely. One of our limitations was that the some of the mentioned tests were not checked in our center; however, according to the patient's negative results of FVL and LA tests, further laboratory tests are not strongly recommended. There is also no Virchow's triad, and examination of the patient's CT findings showed that the patient's splenic vein was open and there was no venous anatomical abnormality. There was no evidence of Budd–Chiari syndrome. Therefore, the specialized team did not consider necessary to perform V G1691A, G20210A, and JAK2V617F tests.

Review of literature did not show any side effects of date seeds. Date seeds powder was used as an herbal medicine in the past to treat diseases such as progeria, anemia, and impotency [23, 24]. Date seeds could be a promising candidate for protection against the CCl4-induced liver intoxication, and this hepatoprotective effect may be attributed to the antioxidant and free radical scavenging activities of some components [25]. Studies have shown that date palm seed consumption has anti-inflammatory [26] and antioxidant properties in humans [27].

El et al. have shown that date seed extract is safe for the liver and kidneys [28]. Therefore, middle-aged women can use it regularly to maintain good health, improve the immune system, and prevent chronic diseases [26]. Clinical implications obtained from the study of Isworo (2020) suggest that physicians may offer palm seed powder as a functional beverage [26]. Alem et al. showed that date seeds could increase the nutritional value of some foods used for human consumption and they could be used as additives in food, pharmaceutical, and cosmetic industries [29]. Some other studies have suggested the possible applications of date seed oil for cosmetics, pharmaceutics, and related products and have less considered the date palm seed for food products [30].

Date seed powder was characterized by low moisture and high carbohydrate and fat contents [31]. Studies have shown that date seeds can be used as a probiotic and interact with the intestinal microflora, especially probiotic bacteria [32]. The results of Darwish et al. have shown that date powder caused the growth of probiotics [33]. On the other hand, the results of Zhu et al. showed an unrecognized mechanistic link between specific dietary nutrients, gut microbes, platelet function, and the risk of thrombosis. Gut microbes can directly contribute to platelet hyperactivity and enhance the thrombotic potential by producing trimethylamine N-oxide (TMAO) [34].

We hypothesize that date seed powder may interact with gut microbiota and cause various reactions producing TMAO and possibly leading to thrombosis. There are complex interactions between diet composition, the gut microbiota, and their metabolites that affect human health [34]. Well-designed clinical trials and further experimental studies are required to determine the influence of probiotics such as date powder on TMAO. Further studies are required due to the lack of evidence, as the review of literature shows insufficient scientific evidence to document and ascertain the (adverse) effects and complications of date seed accurately. Our case report has hypothesized that date seeds can have side effects; however, clinical trials are required to confirm this claim. In addition, most studies have dealt with the health benefits of date seed consumption and have not considered its possible side effects.

## 4. Conclusion

Since date seeds are used in some places for human consumption and disease treatment, it is necessary to pay attention to the side effects and problems associated with their consumption. In geographical areas where date seeds are ingested, health professionals should pay attention to the side effects of date seed, inform consumers, and prevent its complications. The present study can provide valuable and new insights for future studies to better understand and identify the adverse effects and reactions due to date seed intake.
